# Astigmatic traction force microscopy (aTFM)

**DOI:** 10.1038/s41467-021-22376-w

**Published:** 2021-04-12

**Authors:** Di Li, Huw Colin-York, Liliana Barbieri, Yousef Javanmardi, Yuting Guo, Kseniya Korobchevskaya, Emad Moeendarbary, Dong Li, Marco Fritzsche

**Affiliations:** 1 National Laboratory of Biomacromolecules, CAS Center for Excellence in Biomacromolecules, Institute of Biophysics, Chinese Academy of Sciences, Beijing, China; 2 MRC Human Immunology Unit, Weatherall Institute of Molecular Medicine, University of Oxford, Oxford, UK; 3 Kennedy Institute for Rheumatology, University of Oxford, Oxford, UK; 4 Department of Mechanical Engineering, University College London, London, UK; 5 College of Life Sciences, University of Chinese Academy of Sciences, Beijing, China; 6 Rosalind Franklin Institute, Harwell Campus, Didcot, UK

**Keywords:** Biophysical methods, Imaging, Fluorescence imaging, Biophysics

## Abstract

Quantifying small, rapidly progressing three-dimensional forces generated by cells remains a major challenge towards a more complete understanding of mechanobiology. Traction force microscopy is one of the most broadly applied force probing technologies but ascertaining three-dimensional information typically necessitates slow, multi-frame z-stack acquisition with limited sensitivity. Here, by performing traction force microscopy using fast single-frame astigmatic imaging coupled with total internal reflection fluorescence microscopy we improve the temporal resolution of three-dimensional mechanical force quantification up to 10-fold compared to its related super-resolution modalities. 2.5D astigmatic traction force microscopy (aTFM) thus enables live-cell force measurements approaching physiological sensitivity.

## Abstract (web summary)

Quantifying rapidly progressing three-dimensional forces generated by cells remains a major challenge in mechanobiology. Here, the authors show that combining traction force microscopy with astigmatic imaging permits sensitive out-of-plane force estimation on the second timescale.

## Introduction

Cell generated mechanical forces are emerging as a key regulator of biological function^1–3^. Recent evidence indicates that cells modulate their function not only downstream of signalling events triggered by external biochemical stimuli, but that cells employ a diversity of biomechanical mechanisms enabling them to dynamically adjust their mechanics to meet physiological needs^4–6^. Consequently, this forms a previously underappreciated picture wherein cells actively exert and resist biomechanical forces to tune their mechanobiology and thus facilitate their function^7,8^. This is particularly important during the profound three-dimensional (3D) interactions that occur at cell–cell contacts or between cells and their micro-environment. Developing methodologies to accurately quantify cellular forces has therefore become an important mission towards understanding the mechanobiology of cells.

Traction force microscopy (TFM) is perhaps the most widely used mechanical force probing methodology^9–12^, owing to its versatility in mimicking biological and mechanical conditions, ease of implementation, and reliance on widely available materials and equipment^13^. In a typical TFM experiment, a thin (20–30 µm), homogeneous and isotropic, elastic substrate which can either be hydrogels such as polyacrylamide, or silicone-based elastomers such as PDMS, is formed on a glass coverslip^13^. The mechanical and biochemical properties of the gel can be tuned to mimic physiological interactions between the gel surface and the cell by changing its elastic modulus and surface protein functionalisation. When cells interact with the functionalised surface and exert mechanical forces, displacements of fluorescent fiducial markers located at the upper gel layer are imaged using fluorescence microscopy. Fiducial markers can be introduced using randomly dispersed fluorescent beads, or more recently by micro-printing regularly spaced dye molecules onto the substrate facilitating reference-free TFM^14,15^. In two-dimensional TFM (2D-TFM), only the lateral displacements are measured, whereas in two-and-a-half dimensional (2.5D)-TFM, both the lateral and axial components of displacements are measured. TFM can also be performed by embedding the cell within an elastic medium, allowing the induced stresses to be quantified in all three dimensions^16^. In all cases, using a theoretical description of the elastic substrate combined with knowledge of its elastic properties, the inverse problem can be solved and the mechanical forces responsible for the measured displacements can be calculated. Note, the term traction refers to the stresses exerted by the cell at the interface with the gel, while the general term stress is used to describe the 3D stress generated within the bulk of the gel.

TFM allows force measurements whereby the cell can deform the underlying substrate in all spatial dimensions, mimicking biological interactions. This is in contrast to other force quantification modalities such as fluorescent-based force sensors, which while offering single-molecule sensitivity, are not able to capture the full range of biomechanical cellular interactions, for example it currently remains challenging to interpret pushing forces exerted by the cell using DNA-force sensors^17,18^. In addition, because TFM inherently depends on a mechanically deformable substrate, it allows the role of mechanical feedback within the biological system to be investigated.

Despite its importance, quantifying mechanical force production by living cells via TFM remains challenging due to a number of technical limitations. The accuracy and resolution of TFM critically depends on the finite spatio-temporal resolution of the microscopy modality with which the technique is applied^19,20^. The temporal sampling frequency influences the ability to reference and track fiducial markers over time. Consequently, insufficient temporal sampling leads to errors in both the measured magnitude and direction of the applied forces and thus critically influences the sensitivity of TFM experiments. Similarly, the spatial sampling density is constrained by the finite axial and lateral spatial resolution of the optical fluorescence microscope, as the finite size of the point spread function (PSF) resulting from each marker bead imposes an upper limit on the spatial density of beads that can be reliably imaged and tracked within the elastic gel^19^.

Several strategies have been utilised to overcome these limitations, primarily based on its combination with advanced imaging modalities. The use of super-resolution STED (stimulated emission depletion), SIM (structured illumination microscopy), and fluctuation-based SRRF (super-resolution radial fluctuation) microscopy has facilitated the use of increased bead densities, leading to improved accuracy and resolution of force reconstruction^19–22^. However, owing to its reliance on confocal scanning and high illumination powers, STED-TFM is limited to slow acquisition rates, making it ill-suited for force quantification within rapidly evolving biological samples. Because SIM is a wide-field approach using camera acquisition, it provides a more promising route towards rapid force quantification. Indeed, when operating in a 2D, or TIRF mode, SIM-TFM is capable of imaging at both high-spatial and temporal resolution. When combined with 3D-SIM, TFM can operate at high spatial resolution to recover 3D forces, but is restricted in its speed due to the reliance on axial scanning in order to establish the position of the beads in 3D^19^. Elastic resonator interference stress microscopy offers an alternative interferometric approach to measure nano-scale axial forces. However, unlike TFM, determining lateral forces simultaneously remains challenging^23^.

**Fig. 1:**
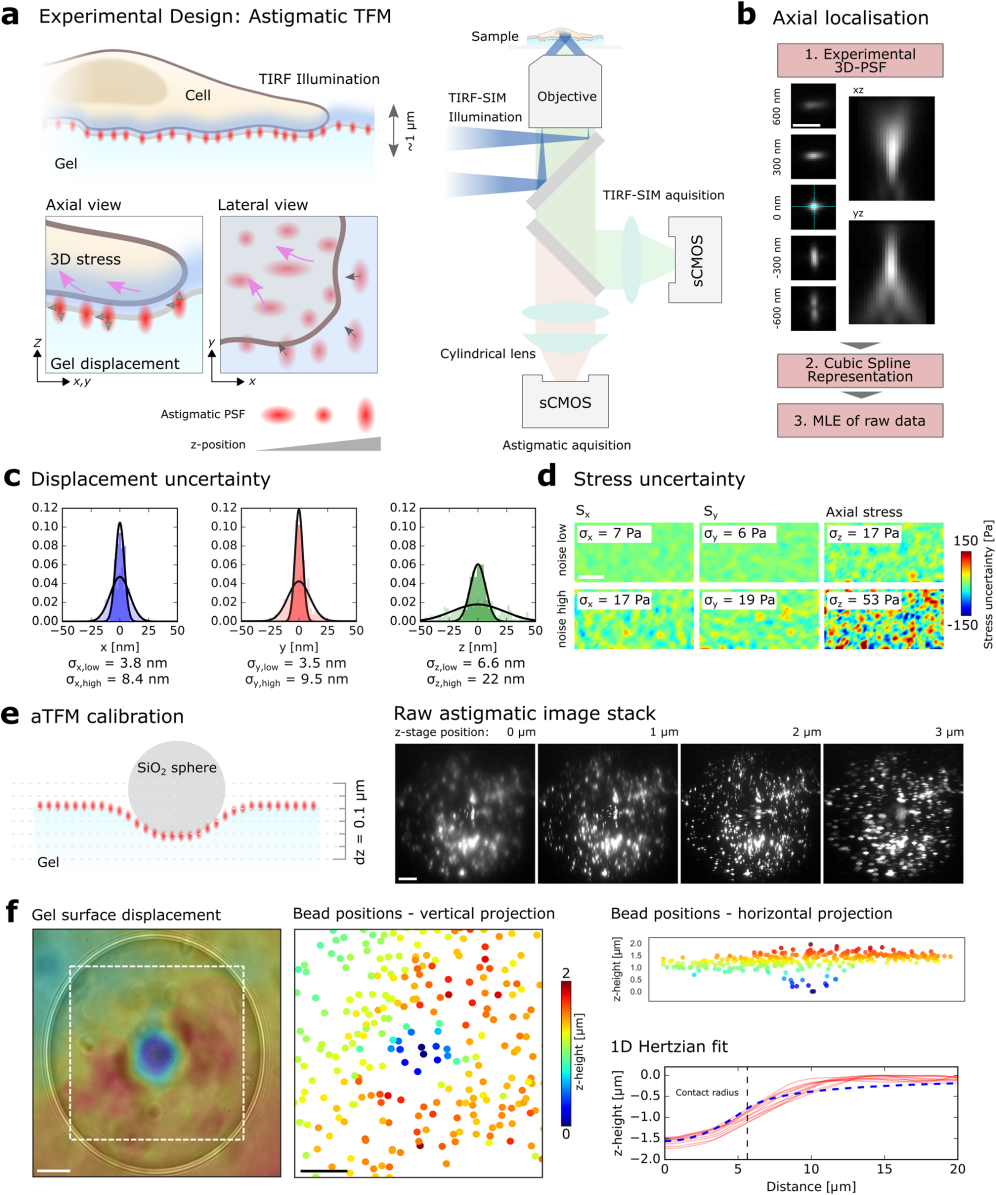
Overview of the uncertainty estimation and calibration of aTFM. **a** Schematic outlining the aTFM method. TIRF illumination at the top surface of the gel excites both cellular structures and fluorescent marker beads. The TIRF-SIM optical path with two sCMOS cameras in combination with the cylindrical lens permits TIRF-SIM imaging of cellular structures while astigmatic imaging of the marker beads allows extraction of axial positional information. **b** Outline of the bead localisation procedure in aTFM. First, the 3D-PSF (point spread function) of the microscope is acquired and modelled using cubic splines. The cylindrical lens in the emission light path introduces an astigmatic modulation of the microscope PSF allowing the orthogonal widths to report on bead position relative to the focal plane. Scale bar is 1 µm. This experimentally acquired PSF model is then compared to the raw bead images and the *z*-position is established via a maximum likelihood estimation (MLE). **c** Estimation of the displacement uncertainty resulting from an MLE of the astigmatic PSF. Plots show the lateral displacements uncertainty (blue and red histograms) as well as the axial displacement uncertainty (green histogram). The displacement uncertainty is calculated for the case of no cellular fluorescent background (low noise) and the case where there is bleed-through of fluorescent signal from the cell (high noise). **d** Estimation of the corresponding uncertainty in the lateral (*S*_*x*_,*S*_*y*_) and axial (*S*_*z*_) stresses for a 10 kPa gel for the case of high and low image noise level. Scale bar is 5 µm. **e** Left: schematic outlining the aTFM calibration using a 70 µm diameter SiO_2_ sphere applying a known force of 3 nN to the upper gel surface. Right: corresponding stack of astigmatic images for different axial locations displaying the degree of PSF aberration resulting from the applied force. Scale bar is 10 µm. **f** Left: Interpolated axial position of the top surface of the gel underlying the SiO_2_ sphere calculated by estimating the position each bead within the FOV. Scale bar is 10 µm. Right: Axially colour coded bead positions within the FOV. Left-bottom: The 1D Hertzian model (dashed blue line) applied to the contact profile (red lines) of the SiO_2_ sphere, allowing the applied force to be estimated.

Despite these technical advances there is an emergent demand for TFM modalities that allow 3D interactions between cells and their environment to be captured at sufficient temporal resolution to allow cellular force probing on the nanometre length-scale, sub-second time-scale, at all three-dimensions of cellular force production^19^. Astigmatic imaging is a technique widely employed in single-molecule localisation microscopy to quantify the 3D position of an emitter relative to the focal plane^24^. By introducing a cylindrical lens directly in front of the camera in the optical path of the emitted light, the microscope’s PSF is reshaped depending on its position relative to the focal plane (Fig. 1a). Crucially, the introduction of astigmatism allows 3D information from a ~1 µm zone surrounding the focal plane to be inferred from a single wide-field image, precluding the need for axial scanning. To this end, the technique allows 3D sampling at single-frame imaging (as fast as 90 ms per SIM image frame) many-fold faster than 3D-SIM. This offers the advantage of increased temporal resolution, but also significantly decreasing the fluorescence excitation light exposure compared to STED and confocal based microscopy, maximising the number of images that can be acquired within a specific time period and minimising photo-bleaching effects.

One drawback of the typical implementation of astigmatic imaging is the need to use epi-illumination such that a sufficient axial volume within the sample is illuminated to allow light from emitters within and outside the focal plane to be collected and their 3D positions established. While this is not typically a problem in localisation microscopy, the use of epi-illumination when coupled to TFM would result in a poor contrast wide-field image of the cell on the gel surface. To address this, we combine astigmatic imaging with total internal reflection fluorescence microscopy (TIRF), reducing the contribution of out-of-plane fluorescence and enhancing image contrast. To enhance the resolution of the cellular image, TIRF is operated in a structural illumination modality using a low numerical aperture illumination (TIRF-SIM) (“Methods”), allowing super-resolution imaging of cellular structures in parallel with a 2.5D force estimation^25^. TIRF illumination can be achieved at the top surface of the TFM gel by matching the refractive index of the gel to the glass below in combination with an oil immersion objective, allowing TIRF-SIM to be performed at the gel-cell interface^26^. Deformations of the interface will result in a shift of the fiducial markers relative to the microscope focal plane, which is registered as an aberrated PSF on the camera. Notably, vertical deformations of the gel surface resulting from cell-generated forces would lead to a small change in the angle of the TIRF incident light at the cell–gel interface. However, provided these deformations remain below a certain limit, the TIRF illumination is maintained (see “Methods”).

Here, to enable sensitive physiological force probing in cells, we combine TFM with fast 2.5D astigmatic imaging and TIRF-SIM (Fig. 1a, b). Applying a combination of top-down sensitivity calibration and live-cell experiments, we quantify the rapid progression of 2.5D cellular force distributions, acquiring at up to 10-fold enhanced temporal resolution owing to single-frame astigmatic imaging rather than multi-frame *z*-stack acquisition. Astigmatic TFM (aTFM) revealed small, rapidly evolving out-of-plane mechanical forces generated during early cell–substrate adherence of cervical cancer cells and initial planar spreading of activating mast immune cells, offering a temporal resolution enhancement and thus enabling cellular force measurements approaching physiological sensitivity.

## Results

With the intent of combining TFM and astigmatic imaging, we introduced a cylindrical lens into the light path of the previously developed super-resolution TIRF-SIM platform. The cylindrical lens was placed in front of the sCMOS camera dedicated to the fluorescent bead channel, allowing the 3D positions of the beads within the gel to be tracked over time^19,25,27^. Simultaneous 2D TIRF-SIM acquisition of the fluorescent cell channel enabled fast mapping of super-resolved cellular dynamics in parallel with 2.5D mechanical force quantification over time (Fig. 1a). Note the introduction of the astigmatism allowed high-resolution axial information to be extracted from the bead images but prohibited the production of a super-resolution SIM bead image.

### aTFM calibration and displacement uncertainty estimation

We first set out to calibrate the dynamic range and sensitivity of aTFM measurements in the absence of cellular forces. To establish the axial dynamic range, using a reference gel sample functionalised with a sparse distribution of fluorescent fiducial marker beads, we calibrated the relationship between the shape of the astigmatic PSF and its position relative to the focal plane. This was achieved by stepping the sample through the focal plane of the microscope using z-piezo scanning while acquiring a single image for each *z*-position, generating a 2 µm stack. Note, a single wide-field astigmatic image of the beads is computed by arithmetic addition of the respective nine individual single TIRF-SIM frames (three angles and three phases) at a frame rate between 1 and 10 fps. Using well-established analysis algorithms^28^, a single experimental PSF was generated by aligning and averaging each individual bead within the acquired stack. Next, applying cubic spline interpolation, an experimentally defined 3D-PSF specific to the optical and experimental setup necessary for aTFM was generated covering the range of 2 µm stack (“Methods”)^26^ (Fig. 1b). Subsequently, the 3D positions and hence displacements of the fluorescent beads could be inferred by performing a maximum likelihood estimation (MLE) between the experimentally defined 3D-PSF and the raw data^28,29^. Note, the above quantitative relationship of PSF shape and *z*-position can be optically controlled by adjusting the distance between the astigmatic lens and the camera in the TIRF-SIM optical path.

To determine the localisation uncertainty of the MLE, we calculated the ensemble standard deviation in the displacement of a subset of beads (unperturbed by any cellular force generation and corrected for 3D drift) relative to their initial position over 100 time points (100 s; see “Methods”). For an area with minimal background intensity the localisation uncertainty was approximately 4 nm in the lateral direction and 7 nm in the vertical direction (Fig. 1c). Introducing a level of image noise equal to that derived from fluorescence bleed-through of the fluorescently labelled cell, the uncertainty increased in all spatial dimensions to approximately 9 nm laterally and 22 nm vertically (Fig. 1c). These values were in line with uncertainty estimations given in 3D super-resolution localisation microscopy^24^. Despite the increase due to the presence of imaging noise, the displacement uncertainty values correspond to a lateral stress uncertainty of approximately 20 Pa and a vertical stress uncertainty of 50 Pa for a 10 kPa gel (Fig. 1d).

Having calibrated the dynamic range and localisation uncertainty of aTFM, we next sought to investigate the method’s ability to experimentally assess 3D deformations under a known static mechanical load. To demonstrate the robustness of the method across an extended axial dynamic range, we applied a total axial force of 3 nN to a soft 250 Pa gel in the form of a 70 µm diameter SiO_2_ sphere and imaged the subsequent displacement field of the fiducial beads (see “Methods”). In this case, because the displacement of the gel surface went beyond the axial dynamic range of a single astigmatic image, we acquired multiple single astigmatic images at 100 nm spacing using piezo scanning (Fig. 1e). By extracting the axial positions of the beads in each frame using the previously outlined localisation procedure and combining with knowledge of the piezo height, it was possible to reconstruct spatial displacement data over a range of 3 µm under the bead, revealing a spherical indentation profile (Fig. 1f). Fitting the SiO_2_ indentation profile with a theoretical Hertzian model led to an estimated axial load of 2.93 ± 0.20 nN (*N* = 3 beads, mean and standard deviation), showing good agreement with the expected value (see “Methods”).

### Quantifying cellular force generation using aTFM

Having established the technical capabilities of aTFM in time-independent control experiments, we next measured the rapid temporal evolution of nano-scale 2.5D mechanical forces in two distinct biological systems: the early adhesion of cervical cancel HeLa cells and the activation of mast immune cells. Crucially, both examples show dynamics on the second time scale making the assessment of axial forces challenging using other imaging modalities. Excitation of the cell’s fluorescent channel (Lifeact-citrine, 516 nm excitation, 529 nm emission) and the fiducial beads (580 nm excitation, 605 nm emission) was implemented using a single 488 nm excitation laser line to maximise the experimental frame-rate (“Methods”).

**Fig. 2:**
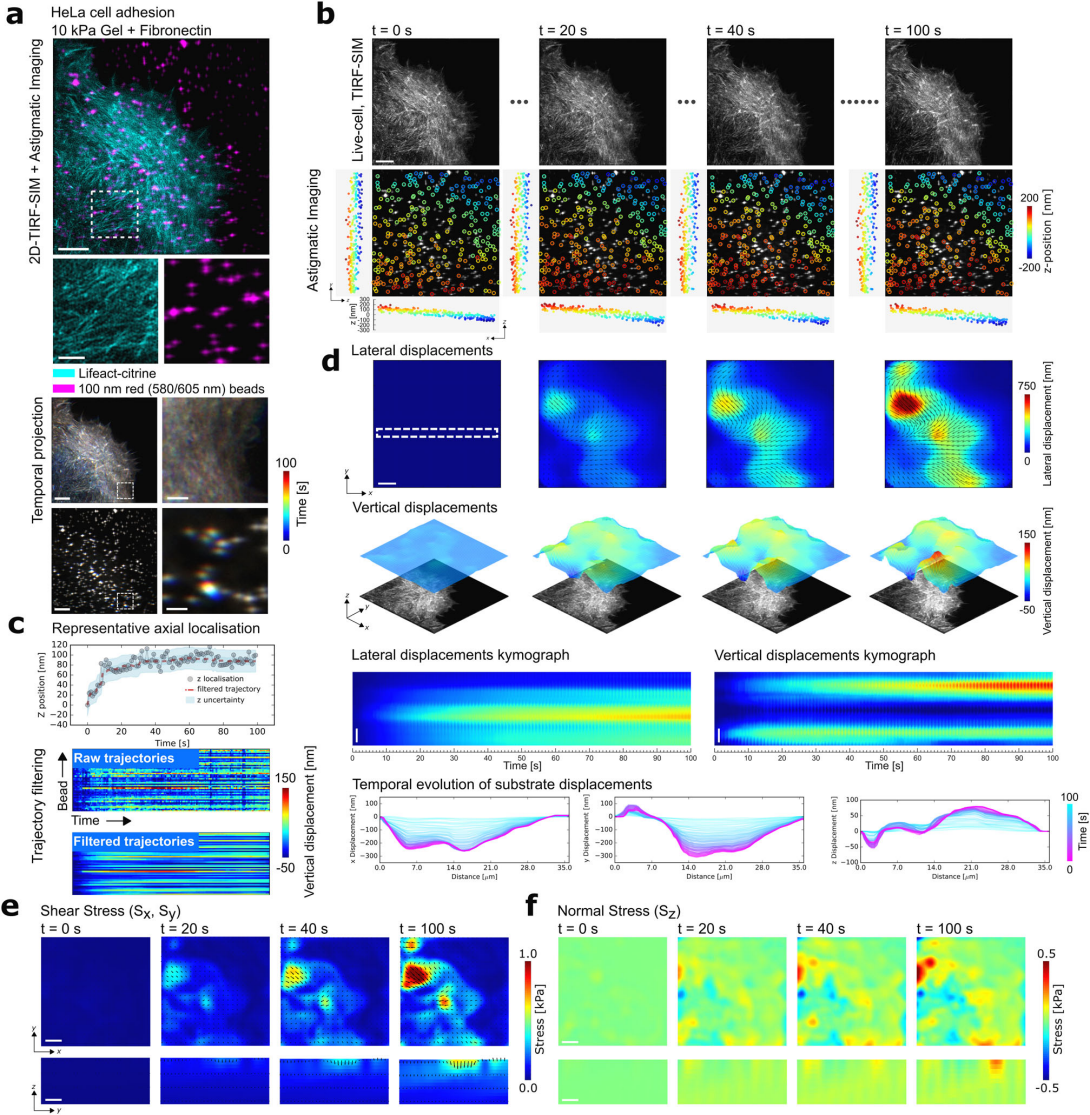
Quantifying out-of-plane force generation during HeLa cell adhesion using aTFM. **a** Upper: TIRF-SIM imaging of a HeLa cell expressing Lifeact-citrine (cyan) adhering to the elastic substrate loaded with fluorescent marker beads (magenta) imaged using the aTFM setup. Scale bar is 5 µm. Lower: colour-coded temporal projection of the TIRF-SIM cell image as well as the astigmatic bead image. Scale bar is 5 µm. Zoom in of dashed box has a scale bar of 1 µm. **b** Upper: TIRF-SIM snap shots of the HeLa cell over the course of the 100 s, 100 frame movie. Lower: Astigmatic bead image overlaid with a scatter plot showing their lateral positions colour coded by their axial positions over time. In addition, the lateral orthogonal projections of the bead positions are plotted. Scale bar is 5 µm. **c** Upper: Plot showing the axial position of a representative raw bead trajectory (grey dots) as well as the filtered position (red) and displacement uncertainty as derived in Fig. 1c (blue region). Lower: Panel showing all unfiltered trajectories (upper) and median filtered trajectories (lower). **d** Upper: Snap shots of the magnitude of lateral displacements resulting from HeLa cell adhesion over the course of the 100 s, 100 frame movie and surface plots of the corresponding vertical displacement of the gel. The cell image is displayed below to facilitate correspondence of displacements with cellular structures. Scale bar is 5 µm. Lower: Both kymographs and line plots showing the continuous temporal evolution of the lateral and vertical surface displacements over the course of the recording. Scale bar is 5 µm. **e** Upper: Snap shots of the magnitude of shear stress resulting from HeLa cell adhesion. Lower: cross-section of the gel showing the distribution of shear stress through the bulk of the gel. Scale bar is 5 µm. **f** Upper: Snap shots of the magnitude of normal stress at the gel surface. Lower: cross-section of the gel showing the distribution of normal stress through the bulk of the gel. Scale bar is 5 µm.

During de novo adhesion, cells have been shown to exhibit predominantly lateral forces^30,31^; nevertheless, recent studies have indicated normal forces may exist that contribute towards the formation of the adhesion sites^32^. To study this phenomenon, we analysed the out-of-plane forces present during early cervical HeLa cancer cell adhesion^19^. Following the previously optimised protocol for HeLa cell adhesion, we first allowed the HeLa cells stably expressing Lifeact-citrine to settle on a 10 kPa silicone substrate functionalised with fibronectin and loaded with red 100 nm fluorescent beads (Fig. 2a)^19^. Ten minutes after deposition of the cells, to capture the continuously forming fresh adhesion contacts of the cell and the associated 3D motions of the fluorescent beads at the top surface of the gel, we simultaneously acquired two colour images using the above described astigmatic TIRF-SIM acquisition scheme. A total of 18 wide-field TIRF images were acquired at each time point (3 angles, 3 phases, and 2 colours on separate cameras with a single excitation laser) at a raw frame rate of 9 frames per second (fps). Subsequent processing of the raw frames produced a TIRF-SIM image of the cellular contact at each time-step (Fig. 2b). Analysing each bead within the area underneath the cell contact with the experimentally acquired PSF using MLE allowed both the lateral and vertical positions of the beads to be extracted over time. Overlapping beads that would prohibit accurate localisation were removed from the analysis and only beads that could be tracked for the duration of the acquisition were included, leading to a final bead density of 0.3 beads per µm^2^. By localising and linking each bead into a trajectory, the displacement of the gel surface relative to the first frame was deduced (“Methods”) (Fig. 2c). To remove spurious localisations, the data were filtered using a temporal median filter with a window size of 10 time points (10 s) and then interpolated in space, applying a spatial median filter with a window size of 10 pixels (0.6 µm) (Fig. 2c).

We observed a gradient of 3D mechanical loading across the field of view (FOV) accompanied by individual adhesion contacts continuously forming and dissipating as the HeLa cell adhered to the surface (Fig. 2d). As expected, as the cell contact matured over the 100 s recording, the gel was displaced laterally towards the cell body, indicative of the cell contractions typically observed during such adhesion processes with lateral displacement magnitudes of 100–750 nm. Intriguingly, accompanying the lateral motions, the gel was also continuously displaced upwards at the cell periphery, while areas away from the edge were displaced downwards with small displacements scaling between 50 and 150 nm (Fig. 2d and Supplementary Movies 1 and 2). These observations were consistent with previous findings showing a rotational moment at the leading edge of an adherent cell^32^. Using a previously established finite element pipeline^19^, both the lateral and vertical displacements were converted to normal (*S*_*z*_) and shear stresses (*S*_*x*,*y*_), providing a 3D map of the overall stress distribution imposed on the gel by the adherent HeLa cell with mechanical stress magnitudes of 0.1–0.5 kPa axially and 0.5–1 kPa laterally (Fig. 2e, f and Supplementary Movies 3 and 4).

The experiments showed that the aTFM modality robustly detected these relatively small vertical displacements on the length scale of a maximum of 150 nm with an uncertainty of 22 nm. For the dual colour aTFM acquisition, a total of 100 images consisting of 1800 frames were acquired (3 phases × 3 angles × 1 *z*-slice × 2 colours at each time point), leading to an overall frame-rate of 1 fps and a total acquisition time of 100 s. Notably, the experimental hardware would allow up to 10 fps with an exposure time of 10 ms provided the biological specimen exhibited a sufficient number of photons.

**Fig. 3:**
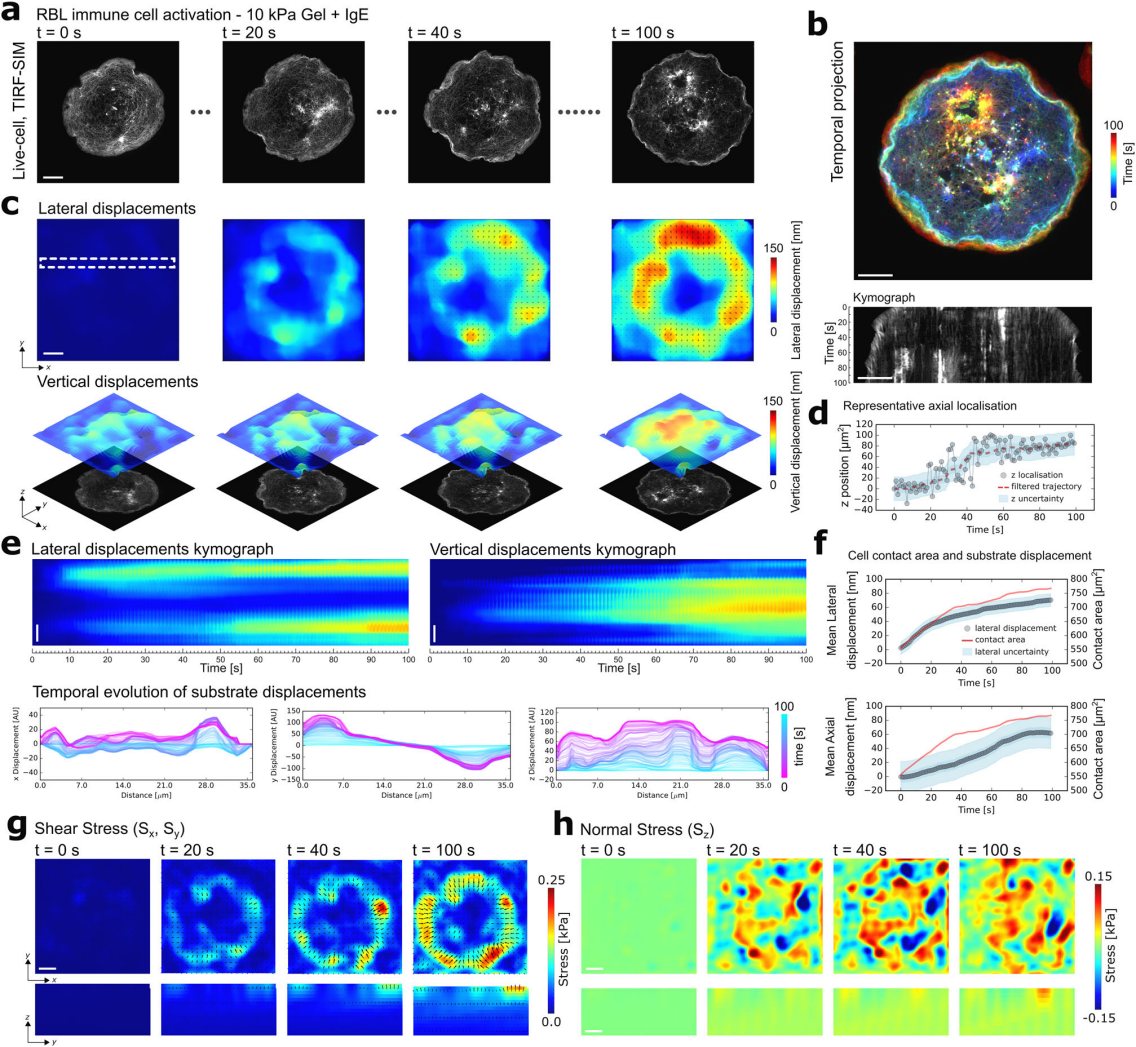
Quantifying out-of-plane force generation during RBL immune cell activation using aTFM. **a** TIRF-SIM snap shots of an activating RBL cell over the course of the 100 s, 100 frame movie. Scale bar is 5 µm. **b** Upper: Temporal projection of RBL cell during activation. Lower: Kymograph indicating the increase in contact area during RBL cell activation. Scale bar is 5 µm. **c** Upper: Snap shots of the magnitude of lateral displacements resulting from RBL cell activation. Lower: Surface plots of the corresponding vertical displacement of the gel. The cell image is displayed below to facilitate correspondence of displacements with cellular structures. Scale bar is 5 µm. **d** Plot showing the axial position of a representative raw bead trajectory (grey dots), as well as the filtered position (red) and displacement uncertainty as derived in Fig. 1c (blue region). **e** Both kymographs and line plots showing the continuous temporal evolution of the lateral and vertical surface displacements over the course of the recording. Scale bar is 5 µm**. f** Plot showing the mean lateral and vertical gel displacements (grey dots) underlying the cell as a function of time during activation overlays with a quantification of cell contact area (red). The corresponding displacement uncertainty as derived in Fig. 1c is shown in blue. **g** Upper: Snap shots of the magnitude of shear stress resulting from RBL cell activation. Lower: Cross-section of the gel showing the distribution of shear stress through the bulk of the gel. Scale bar is 5 µm. **h** Upper: Snap shots of the magnitude of normal stress at the gel surface. Lower: Cross-section of the gel showing the distribution of normal stress through the bulk of the gel. Scale bar is 5 µm.

Next, we quantified 2.5D mechanical force generation during immune cell activation. Immune cell activation depends on the formation of intimate cell–cell contacts and studies have shown that both lateral and axial forces are present during such interactions^7,33,34^. Crucially, such forces have been demonstrated to influence function by perturbing the rates of receptor–ligand binding^7,35,36^. To this end, we studied to distribution of out-of-plane forces present during the activation of the model mast immune cell line rat basophilic leukaemia (RBL) cells^13,20^. Following the previously optimised protocol of RBL cell activation^8^, we allowed the RBL cells stably expressing the Fcε receptor-1 (FcεRI) and F-actin fluorescently labelled with Lifeact-citrine to interact with a 10 kPa silicone substrate coated with the FcεRI-specific antibody IgE and loaded with red 100 nm fluorescent beads at a density of 0.3 beads per μm^2^ (Fig. 3a, b). Applying the same acquisition parameters in both the RBL and HeLa cell experiments, we observed the radial spreading of the RBL cells in the response to IgE-mediated activation. Consistent with the observations in the HeLa cell experiment, we could follow the evolution of the 3D gradient of mechanical loading across the FOV accompanied by individual displacement features at the ventral interface of the RBL cells as they constantly formed fresh adhesion contacts as displayed in the time-lapse images and the respective displacements images (Fig. 3c–e and Supplementary Movies 5 and 6). As the cell spread over the substrate, we detected lateral displacements with magnitudes of 50–150 nm and displacements in vertical direction with magnitudes between 50 and 100 nm. Interestingly, vertical displacements increased most dramatically after the spreading had slowed, indicating a global contraction of the cell following spreading, as has been observed in other immune cells^33^ (Fig. 3f). In addition, the simultaneous TIRF-SIM acquisition revealed that the increase in vertical displacements coincided with a dynamic change in the architecture of the underlying F-actin, with an increasing prevalence of bright sub-diffraction actin patterns (Fig. 3a). This results suggests a change in the actin network mechanics that evolved over time during the activation process^8,37^. Computation of the mechanical forces via finite element calculations revealed magnitudes of normal stresses (*S*_*z*_) between 0.01 and 0.15 kPa and shear stresses (*S*_*x,y*_) between 0.01 and 0.25 kPa, suggesting that the shear forces were stronger than the normal forces generated by activating RBL cells but up to 10-fold smaller compared to those forces measured in HeLa cells (Fig. 3g, h and Supplementary Movies 7 and 8).

## Discussion

Quantifying mechanical force at physiological sensitivity in living cells is vital towards understanding the functional significance of mechanobiology. In this work, we build upon recent advances in cell force quantification by combining TFM and astigmatic imaging. The aTFM methodology provides super-resolved structural information of the living cell, the cell context of force production, as well as the directionality and magnitudes of the respective mechanical forces at increased temporal resolution compared to other fluorescent techniques sensitive to out-of-plane force generation. The usage of astigmatism overcomes the restriction of conventional 2.5D TFM technology to slow multi-frame *z*-stack acquisition with limited sensitivity, precluding the need to scan additional focal planes within the sample. Acquiring a single astigmatic image instead of 100–200 frames at multiple *z*-positions to build 3D force information increases the frame rate, and total imaging duration, while thus minimising the photo-toxicity to the cellular system, as has been quantified in numerous earlier studies^38,39^.

Calibration and control experiments highlighted the experimental power and technical flexibility of aTFM. Experimentally, the method achieved a fitting localisation uncertainty of approximately 9 nm laterally and 22 nm axially, consistent with uncertainty estimations given in 3D super-resolution localisation microscopy^24^. The spatial localisation uncertainty could be further improved by using two separate excitation laser lines sequentially acquiring the cell and bead channels, which was in our setup achieved by the application of a single excitation laser line exciting both the cells expressing Lifeact-citrine and the red fiducial beads to maximise the frame rate of single astigmatic image acquisition. The axial dynamic range of aTFM can be straightforwardly increased by combining astigmatic imaging with piezo z-scanning, but consideration should be given to associated increase in light exposure and decrease in temporal resolution. Alternatively, other mechanisms of introducing PSF reshaping could be explored, for example, double helix^40^ or biplane imaging^41^. This flexibility could prove important when dealing with biological samples producing heterogeneous and/or anisotropic force fields with both small and large mechanical forces simultaneously. Because the astigmatic PSF is enlarged compared to the unaberrated PSF, there is an increased likelihood that neighbouring PSFs may overlap, causing errors in the estimation of the width and hence *z*-position of the fluorescent bead. Care should be taken that the bead density used is optimised to ensure sufficiently high spatial density of displacement information while minimising the number of overlapping PSFs. By acquiring images of the fluorescent beads and the cellular structures on two different cameras, the presented implementation of aTFM permits simultaneous acquisition of a super-resolution SIM image of the cell, facilitating the detection of sub-diffraction cellular structures responsible for force generation. Note, despite the use of TIRF-SIM in this work to enhance the spatial resolution of the cellular imaging, force quantification using aTFM is equally compatible with a conventional TIRF microscope.

Applying aTFM to two different biological cell systems revealed the evolution of mechanical forces over time in adhering HeLa cells and in activating RBL cells, respectively, consistent with previous studies^13,19,20^. The spatio-temporal sensitivity of aTFM allowed the measurement of isotropic and strongly anisotropic 3D stresses. In HeLa cells, shear stresses were almost 10 times the normal stress (*S*_z_ = 0.1–0.5 kPa, 100–500 pN/µm^2^, *S*_*x*,*y*_ = 0.5–1 kPa, 500–1000 pN/µm^2^), while in RBL cells, shear and normal stresses were similar (*S*_z_ = 0.01–0.15, 10–150 pN µm^2^, *S*_*x*,*y*_ = 0.01–0.25 kPa, 10–250 pN/µm^2^).

Future advances in imaging modalities should focus on further improving the sensitivity of TFM, ideally maintaining the 3D information introduced by the astigmatic PSF while increasing the lateral resolution of the astigmatic image. Lateral spatial improvements could, for example, be achieved in the future by applying image analysis algorithms that extract additional spatial information^22^. This would allow both an increased density of displacement information^20^ while taking advantage of the superior axial sensitivity and temporal sampling presented here^19^. In light of these considerations, a straightforward advance of the lateral spatial resolution of TFM ideal for quantifying 2D mechanical forces generated by living cells is the simultaneous acquisition of both the fluorescent cell and bead channels in 2D TIRF-SIM modality while maintaining the extended temporal resolution of aTFM^42^.

## Methods

### Cell culture

RBL-2H3 clone cells (CRL-2256, ATCC, USA; mycoplasma tested) and HeLa cells (product 93021013, Sigma-Aldrich; mycoplasma tested) were cultured at 37 °C in 5% CO_2_. RBL cells were maintained in minimum essential media (MEM) (Sigma Aldrich) containing 15% foetal bovine serum (FBS), 10 mM HEPES (Lonza, UK), 2 mM l-glutamine, and 1% penicillin–streptomycin. HeLa cells were cultured in DMEM (Sigma-Aldrich) supplemented with 10% FBS, 2 mM l-glutamine and 1% penicillin–streptomycin. Cells were split every 2 days at a volume ratio of 1:5. 24 h prior to TFM experiments; RBL cells were treated with 0.05% Trypsin-EDTA (Lonza), facilitating their detachment from the cell culture flask. Cells were then transferred to a rotating chamber at 37 °C in 5% CO_2_ to maintain their suspension state prior to experiments. Stable expression of Lifeact-citrine in both RBL and HeLa cells was achieved via a lentivirus transduction strategy^37^.

### Silicone substrate preparation

The fabrication of the high refractive index silicone gel substrate was adapted from previously published work^26^. To fabricate 10 kPa silicone gels, two elastomer components, QGel 920A and 920B (Quantum Silicones, Richmond, VA) were combined at a 1:1.1 ratio by weight. 250 Pa gels were formed using the same components at a ratio of 1:0.8. After thorough mixing and degassing, 50 µL of the gel solution was pipetted onto an 18 mm #1.5 glass coverslip pre-cleaned using sulfuric acid and hydrogen peroxide and spin coated at 5000 r.p.m. for 10 s to form a 20-µm-thick layer. Gels were cured at 100 °C for 2 h, after which gels were stored in PBS at 4 °C. To attach fluorescent marker beads to the gel surface, the gels were first treated with a 10 % (vol/vol) solution of 3-aminopropyl trimethoxysilane (APTMS) (Sigma-Aldrich, UK) for 5 min. A solution of 100 nm, red fluorescent (580/605) Fluospheres carboxylate-modified microspheres (Invitrogen, UK) at a dilution of 1:1000 was combined with 1-ethyl-3-(3-dimethylaminopropyl) carbodiimide (EDC) (Sigma-Aldrich, UK), in ddH_2_O at a final concentration of 100 µg/mL. The solution was pipetted onto the top surface of the gel at a volume of 100 µL, followed by incubation for 10 min at room temperature. After extensive washing, in the case of HeLa cells the process was repeated with a solution of 1 mg/mL fibronectin in 100 µg/mL of EDC solution (Invitrogen, UK) and incubated at room temperature for a further 10 min. For RBL cells, the gel was first coated in a solution containing 1 mg/mL BSA and 100 µg/mL BSA-TNP and 100 µg/mL EDC. After 1 h incubation at room temperature, the gels were washed in PBS followed by incubation with anti-TNP IgE (Clone IgE-3, BD Biosciences) for a further 1 h.

### TIRF-SIM and astigmatic imaging

TIRF-SIM of low illumination NA, so called GI-SIM, and astigmatic microscopy was performed on a custom-built system controlled by acquisition software written in LabView^25,27^. A single 488 nm excitation laser was used (MPB Communications Inc., 2RU-VFL-P-500-488-B1R) to excite both the Lifeact-citrine channel and the fluorescent beads to facilitate rapid acquisition. The excitation beam was first collimated and passed through the acousto-optic tuneable filter (AOTF, AA Quanta Tech, AOTFnC-400.650-TN). The beam was then expanded and sent to a phase-only modulator, consisting of a polarising beam splitter, an achromatic half-wave plate (HWP, Bolder Vision Optik, BVO AHWP3) and a ferroelectric spatial light modulator (SLM, Forth Dimension Displays, QXGA-3DM). The SLM displays a grating pattern with parameters matching the excitation wavelength, which was used to generate diffraction patterns. The diffraction orders of +1 and −1 were focused on opposite sides of back focal aperture of objective lens (Olympus UAPON 100XOTIRF 1.49NA), and then total internal reflected at the cell–gel interface. The evanescent waves generated by +1 and −1 order light interfered to form the illumination pattern. To maximise the pattern contrast, the diffracted light was maintained with s-polarisation using a polarization rotator. The fluorescent images of Lifeact-citrine and fluorescent beads generated by the applied illumination pattern were collected by the same objective, separated by a dichroic beam splitter (Chroma, ZT405/488/560/647tpc), and focused by respective tube lens onto corresponding sCMOS camera (Hamamatsu, Orca Flash 4.0 v3). A cylindrical lens (Optosigma, CLB-3030-1000PM) was introduced in front of the camera to generate astigmatic imaging of the marker beads. For each frame, the acquisition time was 10–100 ms, leading to a super-resolution image (three angles and three phase, nine frames total) every 90–900 ms calculated using a reconstruction method described previously^27,43^. For live-cell imaging, a micro-incubator (H301, Okolabs, Naples, Italy) at 37 °C and 5% CO_2_ was used.

During the aTFM acquisition, the TIRF interface at the top of the silicone gel is displaced in the axial direction due to cell generated normal stresses. This will lead to a change in the angle of incidence relative to the gel–sample interface. For the acquired data, the dynamic range of axial displacement was of the order of 500 nm. If this is assumed to act over the contact area of the cell (15 µm), this equates to an angular distortion of the gel of ~1.5°. The excitation light has an incident NA ranging from 1.38 to 1.41; therefore, the incident angle necessary to achieve TIRF illumination was sin^−1^(1.41/1.515) = 68.5°. If the surface deviates from being flat, the incident angle is 68.5° ± 1.5°, giving a corresponding range in NA from 1.395 to 1.42. TIRF illumination for the described setup is typically achieved for NA > 1.38, indicating that TIRF illumination is maintained during deviations from a flat surface inherent to the aTFM acquisition.

### 3D localisation calibration and displacement quantification

To establish the shape of the astigmatic PSF as a function of its position relative to the focal plane, an experimentally acquired astigmatic PSF was modelled using cubic splines, as outlined in ref. ^28^. To this end, a 2 µm *z*-stack with 10 nm step size was acquired of sparsely distributed fluorescent beads on the top surface of the silicone gel substrate. Using the MATLAB-based software routine (calibrate3D_GUI.m, https://github.com/jries/fit3Dcspline), a single 3D PSF stack was formed by aligning and averaging each of the beads present in the image, followed by a cubic spline interpolation. The cubic spline interpolated PSF serves an experimentally acquired model PSF that can then be compared to the data in order to establish the *z*-position of the florescent beads. Owning to the consistent gel thickness used throughout the experiments, this calibration was performed for a representative gel, and the acquired experimental PSF applied to all subsequent data sets.

In order to localise the 3D spatial position of each of the beads within the aTFM acquisition, we utilised the MATLAB-based software routine (simplefitter_GUI.m, https://github.com/jries/fit3Dcspline) together with the PSF model acquired from the calibration. The software estimates the position of each of the fluorescent beads within the astigmatic image by comparing the raw data to the experimentally acquired model PSF using MLE.

Following localisation, the bead positions were linked into trajectories using the Python single-particle tracking library known as trackpy (https://soft-matter.github.io/trackpy). The ensemble of trajectories was then corrected for 3D sample drift using a sub-set of reference beads far away from the cell contact. Trajectories were then filtered using a median filter (width = 10 frames) to remove spurious localisations that would lead to erroneous measure of gel displacement. A final displacement field at each time point was produced by interpolating the individual bead displacements onto a regular grid, followed by a spatial median filter of width 10 pixels (604 nm).

### aTFM calibration

To demonstrate aTFM using a known static mechanical load, SiO_2_ spheres (63–75 μm, Tianjin BaseLine, Unibead 6-7-6000, density = 2.65 g/cm^3^) were deposited on a 250 Pa silicone substrate loaded with red 100 nm fluorescent beads. First, the diameter of the individual SiO_2_ spheres was measured by fitting the full-width of half-maximum (FWHM) of SiO_2_ sphere in a bright-field image. Second, a 3 µm z-stack of astigmatic images of the 100 nm fluorescent beads was acquired by stepping the axial piezo in 100 nm intervals through the SiO_2_ contact area. Third, a spherical indentation profile was reconstructed by combining the localised axial position and the piezo height. Finally, the indentation force, *P*, was estimated by fitting the SiO_2_ indentation profile with a theoretical Hertzian model^44^ for the normal displacement of the gel surface, *u*_*z*_ as a function of the distance from the centre of the contact, *r*, for a gel of elastic modulus, *E*, and contact radius, *a*:

1 $$u_z\left( r \right) = \frac{{3P}}{{4Ea}}\left( {1 - \frac{{r^2}}{{2a^2}}} \right)$$

Inside the contact region (*r* < *a*), and,

2 $$u_z\left( r \right) = \frac{{3P}}{{4Ea}}\frac{1}{\pi }\left\{ {\left( {2 - \frac{{r^2}}{{a^2}}} \right)\sin^{ - 1}\left( {\frac{a}{r}} \right) + \frac{r}{a}\sqrt {1 - \frac{{a^2}}{{r^2}}} } \right\}$$

Outside the contact region (*r* > *a*).

### Mechanical force quantification via finite element simulations

A commercial finite element software (ABAQUS, Dassault Systèmes) was used to simulate the mechanical interaction between the cell and the substrate. A detailed description of the finite element formulation is provided in a previous publication^19^. Briefly, a cuboid section of the substrate was modelled and the domain size selected to be large enough such that the deformations induced by the applied stress field did not reach the borders (semi-infinite condition). Quadratic tetrahedral 10-node elements (C3D10M) with second-order accuracy were employed to mesh the domain and a linear elastic constitutive model was used to describe the mechanical behaviour of the substrate and elements were assumed to be nearly incompressible (Poisson’s ratio 0.495). Since the displacement vectors were only known at the locations of fluorescent beads, a cubic interpolation scheme was used to estimate and map components of displacement vectors at nodes of the structured finite element mesh on the top surface of the gel. The components of stress tensors and deformation vectors were extracted from output files (.odb file) using a custom-written Python script. An image processing toolbox (MATLAB 2020a, Mathworks) was employed in combination with ImageJ to display stress/displacement fields in the region beneath the cell.

### Statistics and reproducibility

aTFM calibration experiments using SiO_2_ spheres were performed using *N* =  3 spheres on three independent gels. For aTFM experiments on both HeLa and RBL cells, force data were acquired in at least 20 cells in three independent experiments in each case, of which representative data are presented in Figs. 2 and 3.

### Reporting summary

Further information on research design is available in the Nature Research Reporting Summary linked to this article.

## Supplementary information

Description of Additional Supplementary Files

Supplementary Movie 1

Supplementary Movie 2

Supplementary Movie 3

Supplementary Movie 4

Supplementary Movie 5

Supplementary Movie 6

Supplementary Movie 7

Supplementary Movie 8

Reporting Summary

## Data Availability

The data that support the findings of this study are available from the corresponding author upon reasonable request.
