# Supplementary material for: Astigmatic traction force microscopy (aTFM)
Source: Nat Commun. 2021 Apr 12;12:2168. doi: 10.1038/s41467-021-22376-w (PMC8042066; doi:10.1038/s41467-021-22376-w)
Supplement: Supplementary file 1 — Description of Additional Supplementary Files [file 41467_2021_22376_MOESM1_ESM.pdf]

Description of Additional Supplementary Files

File Name: Supplementary Movie 1
Description: Evolution of the lateral displacements of the gel substrate resulting from the adhesion of a HeLa cell to the top surface of the silicone gel. Heat-map shows displacements overlaid with the TIRF-SIM image of the Lifeact-citrine labelled cell. Total movie duration is 100s. Scale bar is 5 µm.

File Name: Supplementary Movie 2
Description: Evolution of the vertical displacements of the gel substrate resulting from the adhesion of a HeLa cell labelled with Lifeact-citrine to the top surface of the silicone gel. Total movie duration is 100s.

File Name: Supplementary Movie 3
Description: Evolution of the shear stress applied by the adherent HeLa cell to the top surface of the silicone gel. Total movie duration is 100s. Scale bar is 5 µm.

File Name: Supplementary Movie 4
Description: Evolution of the normal stress applied by the adherent HeLa cell to the top surface of the silicone gel. Total movie duration is 100s. Scale bar is 5 µm.

File Name: Supplementary Movie 5
Description: Evolution of the lateral displacements of the gel substrate resulting from the activation of an RBL cell labelled with Lifeact-citrine at the top surface of the silicone gel. Heat-map shows displacements overlaid with the TIRF-SIM image of the Lifeact-citrine labelled cell. Total movie duration is 100s. Scale bar is 5 µm.

File Name: Supplementary Movie 6
Description: Evolution of the vertical displacements of the gel substrate resulting from the activation of an RBL cell labelled with Lifeact-citrine at the top surface of the silicone gel. Total movie duration is 100s.

File Name: Supplementary Movie 7
Description: Evolution of the shear stress applied by the activating RBL cell at the top surface of the silicone gel. Total movie duration is 100s. Scale bar is 5 µm.

File Name: Supplementary Movie 8
Description: Evolution of the normal stress applied by the activating RBL cell at the top surface of the silicone gel. Total movie duration is 100s. Scale bar is 5 µm.
